# Use superb microvascular imaging to diagnose and predict metastatic cervical lymph nodes in patients with papillary thyroid carcinoma

**DOI:** 10.1007/s00432-024-05770-x

**Published:** 2024-05-21

**Authors:** Ting Huang, Pin-Tong Huang, Zhi-Yan Luo, Ji-Fang Lv, Pei-Le Jin, Tao Zhang, Yu-Lan Zhao, Yong Wang, Yu-Rong Hong

**Affiliations:** 1 Department of Ultrasound in Medicine, The Second Affiliated Hospital of Zhejiang University School of Medicine, Hangzhou, China; 2 Department of Thyroid Surgery, The Second Affiliated Hospital of Zhejiang University School of Medicine, Hangzhou, China

**Keywords:** Superb microvascular imaging, Conventional ultrasound, Metastatic cervical lymph node, Papillary thyroid carcinoma, Nomogram

## Abstract

**Purpose:**

Papillary thyroid carcinoma (PTC) with metastatic lymph nodes (LNs) is closely associated with disease recurrence. This study accessed the value of superb microvascular imaging (SMI) in the diagnosis and prediction of metastatic cervical LNs in patients with PTC.

**Methods:**

A total of 183 cervical LNs (103 metastatic and 80 reactive) from 116 patients with PTC were analysed. Metastatic cervical LNs were confirmed by pathology or/and cytology; reactive cervical LNs were confirmed by pathology or clinical features. The characteristic of conventional ultrasound (US) was extracted using univariate and multivariate analyses. The diagnostic performance of US and SMI were compared using the area under the receiver operating curve (AUC) with corresponding sensitivity and specificity. A nomogram was developed to predict metastatic LNs in patients with PTC, based on multivariate analyses.

**Results:**

L/S < 2, ill-defined border, absence of hilum, isoechoic or hyperechoic, heterogeneous internal echo, peripheral or mixed vascular pattern on color Doppler flow imaging (CDFI) and SMI, and a larger SMI vascular index appeared more frequently in metastatic LNs in the training datasets than in reactive LNs (*P* < 0.05). The diagnostic sensitivity, specificity and accuracy of SMI vs US are 94.4% and 87.3%, 79.3% and 69.3%, and 87.6% and 79.1%, respectively; SMI combined with US exhibited a higher AUC [0.926 (0.877–0.975)] than US only [0.829 (0.759–0.900)]. L/S < 2, peripheral or mixed vascular type on CDFI, and peripheral or mixed vascular types on SMI were independent predictors of metastatic LNs with PTC. The nomogram based on these three parameters exhibited excellent discrimination, with an AUC of 0.926.

**Conclusion:**

SMI was superior to US in diagnosing metastatic LNs in PTC. US combined with SMI significantly improved the diagnostic accuracy of metastatic cervical LNs with PTC. SMI is efficacious for differentiating and predicting metastatic cervical LNs.

**Supplementary Information:**

The online version contains supplementary material available at 10.1007/s00432-024-05770-x.

## Introduction

Papillary thyroid carcinoma (PTC) is considered as an indolent carcinoma with a favorable prognosis, and patients with PTC have long-term survival (Haugen et al. 2016). Nevertheless, approximately 30–50% of patients with PTC have metastatic cervical lymph nodes (LNs) on preoperative ultrasonography, which is related to an increased recurrence rate (Scheumann et al. 1994; Mao et al. 2020; Mansour et al. 2018). Radical dissection of lymph node metastases results in better survival and a lower recurrence rate (Scheumann et al. 1994). However, performing prophylactic central cervical lymph node dissection (CLND) in patients with PTC without clinically involved central cervical lymph node metastases is controversial (Haugen et al. 2016). Accurate detection of metastatic cervical LNs before surgery is essential to seek optimal clinical treatment and avoid unduly prophylactic CLND.

As the primary imaging examination method for detecting cervical LNs, conventional ultrasound (US) is widely used to distinguish between malignant and benign LNs (Haugen et al. 2016). Sonographic features, including hilar absence, round shape, hyperechogenicity, cystic component, microcalcification, and peripheral vascular pattern, were considered critical signs for predicting metastatic LNs (Bayramoglu et al. 2018; Ahuja and Ying 2003; Leenhardt et al. 2013). Previous studies have indicated that preoperative US demonstrates poor sensitivity in the diagnosis of metastatic cervical LNs, especially the central LNs (Khanna et al. 2011; Zhao and Li 2019; Khokhar et al. 2015; Lee et al. 2015). The overlap of benign and malignant LNs on ultrasonography limits the application of conventional US (Guo et al. 2022; Zhang et al. 2021). In addition, color Doppler flow imaging (CDFI) cannot identify aberrant blood vessels at low speeds in malignant lesions (Bonacchi et al. 2017).

Contrast-enhanced ultrasound (CEUS) can detect smaller blood vessels and provide detailed perfusion information (Lekht et al. 2016). Our previous study indicated that CEUS significantly improved the diagnostic sensitivity and accuracy compared to US in metastatic LNs (Hong et al. 2017). Nevertheless, CEUS is invasive and expensive, and occasional anaphylactic reactions caused by the contrast agent are also a problematic issue.

Superb microvascular imaging (SMI) is an emerging technique for vascular imaging based on Doppler technology. SMI can detect tiny blood flow and filter clutter waves to reveal the real-time blood flow in malignant lesions (Fu et al. 2021). Several previous studies have indicated the advantage of SMI in differentiating benign and malignant cervical LNs compared to conventional US and power Doppler imaging (Sim et al. 2019; Ryoo et al. 2016; Seongyong et al. 2020; Muhammad et al. 2022). However, to the best of our knowledge, this study is the first to explore the diagnostic value of SMI in discriminating cervical metastatic LNs with PTC. We believe that SMI is superior to conventional US in distinguishing between metastatic and benign LNs.

In the current study, we used SMI to assess blood flow distribution in cervical LNs with PTC, and compared the blood flow characteristics between metastatic and reactive LNs to evaluate the diagnosis accuracy. Additionally, we developed and validated a nomogram to predict metastatic LNs based on combined US and SMI features.

## Materials and methods

### Patients

**Fig. 1:**
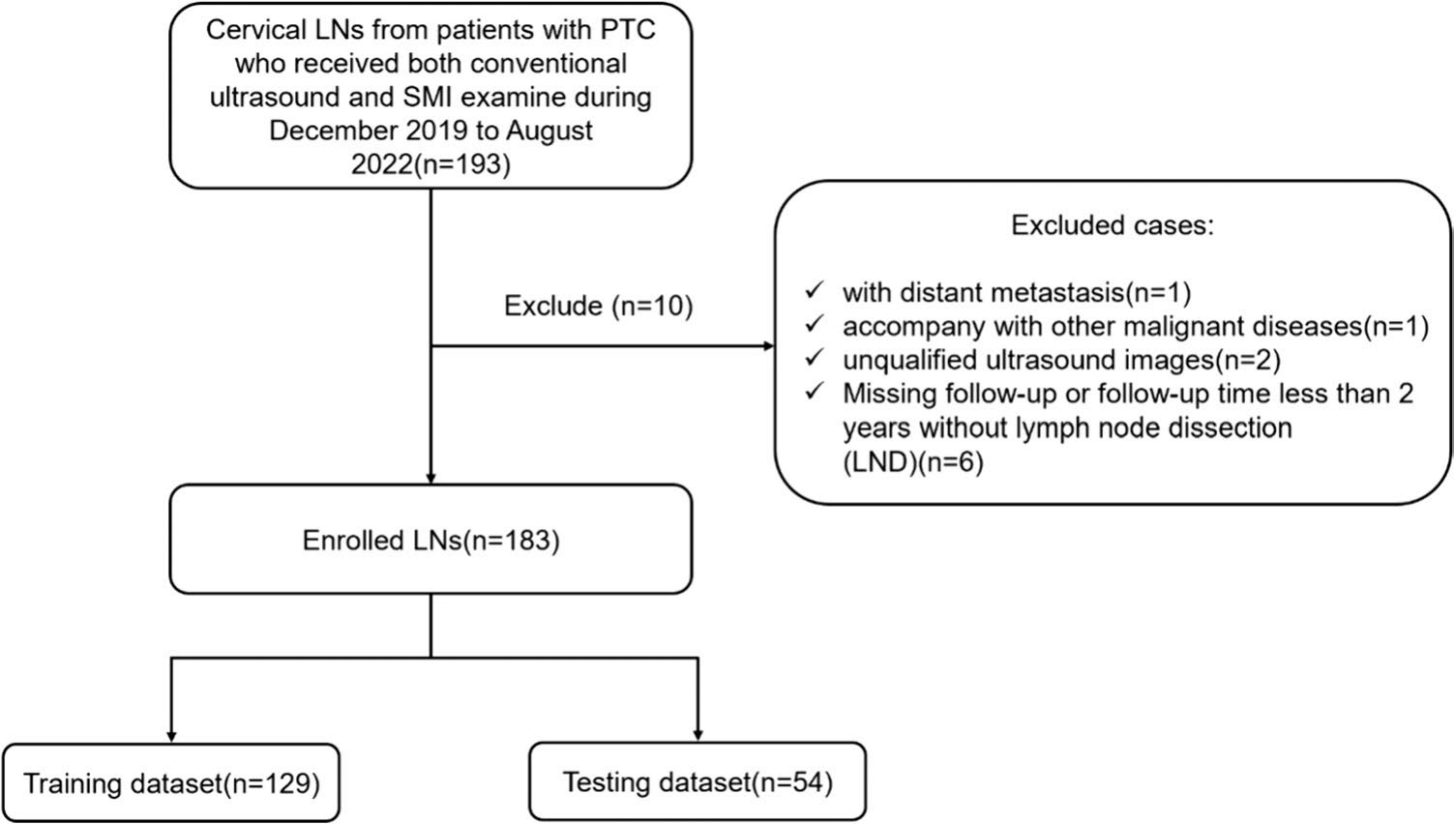
Flowchart of patient selection for differentiating metastatic LNs from patients with PTC. *PTC* papillary thyroid carcinoma, *SMI* superb microvascular imaging, *LN* lymph node

This study was approved by the Institutional Review Board of Second Affiliated Hospital of Zhejiang University School of Medicine (the approval number from the Ethics Committee is 2022-0879). The requirement for Informed consent was waived for this retrospective study. Patients with PTC who underwent both conventional US and SMI examination in the cervical LNs region between December 2019 and August 2022 were selected for this study. The inclusion criteria of the LNs were as follows: (1) metastatic LNs confirmed by a histopathologic examination or cytology; (2) reactive LNs confirmed as benign lesions by histopathologic examination/cytology or < 20% increase in LNs diameter size measured by US at least 2 years follow-up after thyroidectomy for thyroid cancer. (3) US and SMI were performed in the Department of Ultrasound within 2 weeks before surgery or/with fine needle aspiration (FNA); (4) complete medical information available; The exclusion criteria were as follows: (1) patients with distant metastasis (n = 1); (2) patients with other malignant diseases (n = 1); (3) unqualified ultrasound images (n = 2); (4) missing follow-up or the follow-up period less than 2 years without lymph node dissection (LND) (n = 6). Finally, 103 metastatic LNs (54 patients) and 80 reactive LNs (62 patients) were included. A flowchart of patient enrollment is shown in Fig. 1.

### Ultrasound examination

The US diagnostic apparatus was Aplio 900 (Canon Medical Systems, Tokyo, Japan) equipped with a linear array i18LX5 transducer for gray-scale US, CDFI and SMI. SMI was performed in the monochromatic mode, and the SMI parameters were set as a low velocity range (1.0–1.5 cm/s) and high frame rate (25–30 frames/s) according to the manufacturer’s recommendations. The color gain was maximized based on invisible background noise (41 dB).

Patients were put on a bed in the supine position with the neck fully exposed during conventional US and SMI examination. US and SMI examination were operated by two head and neck clinical radiologists with > 10 years of experience in thyroid ultrasound diagnosis. The sonographic characteristic of cervical LNs, including location, size, shape, border, hilum of lymph, echogenicity, internal echo pattern, cystic necrosis, calcification, vascularity pattern of CDFI, vascularity index of CDFI, vascularity pattern of SMI, vascularity index of SMI, were carefully recorded during the examination.

### Image analysis

**Fig. 2:**
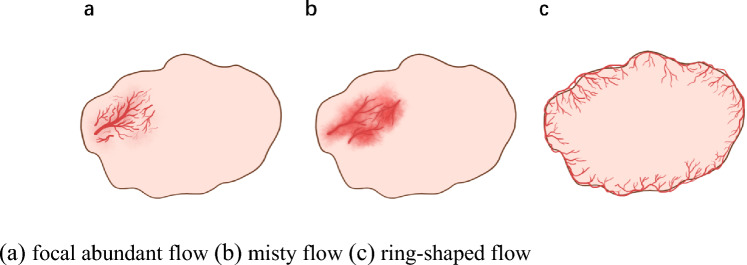
**a** focal abundant flow, **b** misty flow, **c** ring-shaped flow

Two experienced radiologists (6 and 25 years, respectively) who were blinded to the patients’ clinical information reviewed all the US and SMI images. If there was a disagreement between the two radiologists, it was discussed until a unified conclusion was achieved. The location was classified as either central cervical (levels VI–VII) or lateral cervical (levels I–V) according to the American Thyroid Association Surgery Working Group. The long diameter (L) and short diameter (S) were measured as the maximum and vertical diameter of the LN, respectively, and L/S was calculated. L/S was divided into two groups (L/S ≥2, and L/S < 2). The shapes were classified as irregular or oval. The border of LNs was designated as well-defined or ill-defined. Echogenicity was classified as hypoechoic, isoechoic, or hyperechoic compared to the adjacent muscle tissue. The internal echo patterns were heterogeneous or homogeneous. The presence or absence of a hilum, cystic necrosis, and calcification were also recorded. Calcification was divided into microcalcification and macrocalcification. On both CDFI and SMI, the vascular pattern of the lymph node was classified as follows: (1) avascular type, no vessel within the LNs; (2) hilar type, a central vessel with/without branches; (3) peripheral type, vessels running along the capsule without hilar vessel; (4) mixed type, peripheral types coexist with hilar types (including abundant, chaotic blood flow) [16]. The vascular indices of CDFI and SMI were classified into four levels as follows: (1) level 0: no vessel signal detected in LNs; (2) level 1: less than 1/3 area of LN occupied by vascular signal; (3) level 2: area of vascular signal occupied between 1/3 and 2/3 of the LN area; and (4) level 3: more than 2/3 area of LN occupied by vascular signal. According to our observation, several special vascular subtypes extended from peripheral type and mixed type appeared in partial metastatic LNs particularly. The concepts of these special vascular subtypes were explained as follows (Fig. 2):

1. Focal abundant flow: abundant, chaotic vessels aggregate in one/several areas.
2. Misty flow: abundant tiny blood vessels aggregate in focal area, which resemble a mist.
3. Ring-shaped flow: dense vessels running along capsule, appearing like a ring.

### Lymph node localization

The location, shape and size of the LNs were considered to match pathological examinations with US. A mark was drawn on corresponding area vertically to the skin according to US examination again one day before the LND. Matched LNs on both US and pathological examinations were included in this study.

### Statistical analyses

The R studio software (version 4.2.1) and SPSS statistical software (version 24.0; IBM, USA) were used to analyse the data. Continuous variables were described as mean ± standard deviation (SD) and analysed by *t* test. Pearson’s Chi-square test or Fisher’s exact test was used to compare categorical variables. Receiver operating characteristic (ROC) curve analysis was performed to compare the discriminative abilities of US and SMI. The optimal cut-off value was determined using the Youden index. Sensitivity, specificity, positive predictive value, negative predictive value, and accuracy were calculated. Multivariate logistic regression was performed on the variables which proved to be statistically significant on univariate analysis. Statistical significance was set at *P* < 0.05.

A prediction model was built based on the multivariate logistic regression to predict metastatic LNs. The R software was used to construct a nomogram to predict the risk of metastatic LN with PTC. The ROC curve was used to evaluate the diagnostic performance of the model with the corresponding package.

## Results

### Demographic and clinicopathological data

There were 103 metastatic LNs from 54 patients and 80 reactive LNs from 62 patients, which were randomly divided into testing and training datasets (*n* = 54 and *n* = 129, respectively) (Table S1). Of the 103 metastatic LNs, five were confirmed by cytology, and 98 were diagnosed by histopathology. There were no significant differences between the training and testing datasets in terms of clinical features, including sex, age, or LN location (all *P* > 0.05).

### Univariate and multivariate analyses of clinical information, sonographic and SMI features

The US and SMI features of the LNs are described in Table S1. There was no significant difference between the training and testing datasets in terms of US or SMI features (all *P* > 0.05), except for size (*P* = 0.044). Univariable analysis of the training cohort showed significant difference between reactive LNs group and metastatic LNs group in ratio (L/S), border, hilum, echogenicity, homogeneity, CDFI vascular pattern, SMI vascular pattern and SMI vascular index (*P* < 0.05 for all), as shown in Table 1. These results were subsequently incorporated into the multiple logistic regression model.

**Table 1:** Univariate analysis of demographic and sonographic data in the training cohort

Parameter	Reactive LN (n = 58)	Metastatic LN (n = 71)	*P* value
Sex			0.989
Male	41 (71)	49 (69)	
Female	17 (29)	22 (31)	
Age	38 ± 11	38 ± 12	0.589
Location			0.187
Lateral	46 (80)	49 (69)	
Central	12 (20)	22 (31)	
Size(mm)	12.33 ± 4.52	11.47 ± 4.91	0.636
Ratio(L/S)			0.011
≥ 2	43 (74)	36 (51)	
< 2	15 (26)	35 (49)	
Shape			0.137
Irregular	30 (52)	47 (66)	
Oval	28 (48)	24 (34)	
Border			0
Ill-defined	15 (26)	40 (56)	
Well-defined	43 (74)	31 (44)	
Hilum			0.001
Present	39 (67)	65 (92)	
Absent	19 (33)	6 (8)	
Echogenicity			0
Isoechoic or hyperechoic	20 (34)	52 (73)	
Hypoechoic	38 (66)	19 (27)	
Homogeneity			0
Homogeneous	36 (62)	14 (20)	
Heterogeneous	22 (38)	57 (80)	
Cystic necrosis			0.064
Present	0 (0)	5 (7)	
Absent	58 (100)	66 (93)	
Calcification			0.094
Absent or macrocalcification	51 (88)	53 (75)	
Microcalcification	7 (12)	18 (25)	
CDFI vascular pattern			0
Avascular or hilar type	48 (83)	19 (27)	
Peripheral or mixed type	10 (17)	52 (73)	
CDFI vascular index			0.104
0	17 (29)	13 (18)	
1	38 (66)	46 (65)	
2	3 (5)	10 (14)	
3	0 (0)	2 (3)	
SMI vascular pattern			0
Avascular or hilar type	46 (79)	4 (6)	
Peripheral or mixed type	12 (21)	67 (94)	
SMI vascular index			0
0	2 (3)	3 (4)	
1	43 (74)	28 (40)	
2	12 (21)	23 (32)	
3	1 (2)	17 (24)	

*LN* lymph node, *CDFI* color Doppler flow imaging, *SMI* superb microvascular imaging

After multivariate analysis, L/S < 2, peripheral or mixed vascular type on CDFI and peripheral or mixed vascular type on SMI were selected as independent predictors of metastatic LNs in PTC (*P* < 0.05, Table 2). A peripheral or mixed-type SMI vascular pattern was the best predictor of metastatic LNs, with an odd ratio (OR) of 40.83.

**Table 2:** Multivariate analysis of risk variables for metastatic LN of PTC

差Characteristic	OR	Odds ratio (95% CI)		*P* value
L/S < 2	8.684	2.276	45.651	0.004
Peripheral or mixed type of CDFI	5.982	1.624	23.695	0.008
Peripheral or mixed type of SMI	40.829	11.092	211.476	0.000
Constant	0.024	0.004	0.087	0.000

*LN* lymph node, *PTC* papillary thyroid carcinoma, *OR* odds ratio, *CI* confidence intervals, *CDFI* color Doppler flow imaging, *SMI* superb microvascular imaging

### ROC analysis

**Fig. 3:**
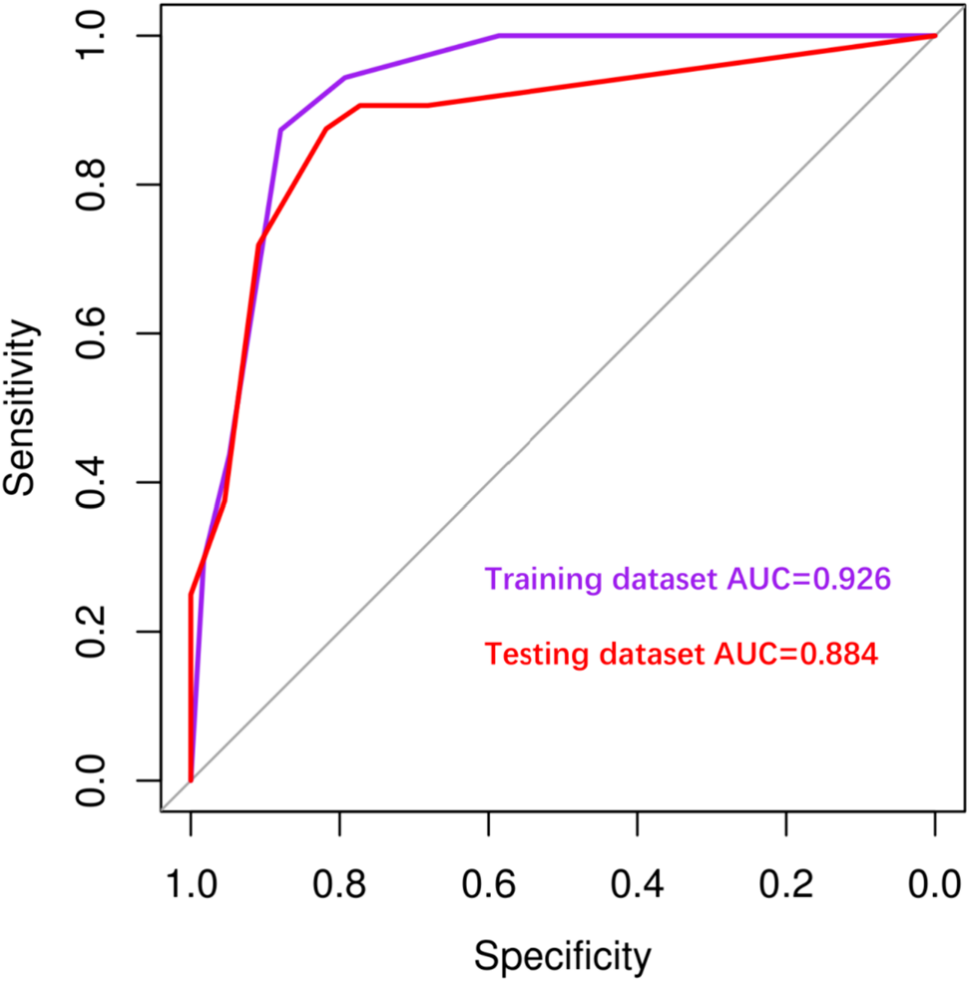
ROC curve analysis for predicting the probability of metastatic LNs in training cohort (AUC = 0.926) and testing cohort (AUC = 0.884); AUC: area under curve

Parameters with statistical significance between metastatic and reactive LNs in training datasets were selected to analyse the sensitivity, specificity, positive predictive value (PPV), negative predictive value (NPV) and accuracy of US, SMI, and SMI + US (Table 3). As shown in Table 3, SMI can improve the diagnostic sensitivity, PPV, NPV and accuracy notably in contrast to US alone. SMI combined with US can improve the specificity and PPV compared to both US and SMI solely, with the highest area under curve (AUC) (0.926, 95% CI 0.877–0.975). The same results were observed in the ROC curve (Fig. S1). The ROC curve analysis for predicting the probability of metastatic LNs in the training and testing cohorts is shown in Fig. 3. This demonstrated consistency in the training and testing datasets for predicting metastatic LNs.

**Table 3:** Diagnostic accuracy of US, SMI, and US + SMI for metastatic LNs in the training cohort

	AUC	Sen (%)	Spe (%)	PPV (%)	NPV (%)	Acc (%)
US	0.829 (0.759–0.900)	87.3	69.3	77.5	81.6	79.1
SMI	0.868 (0.799–0.938)	94.4	79.3	84.8	92.0	87.6
SMI + US	0.926 (0.877–0.975)	87.3	87.9	89.9	85.0	87.6

*AUC* area under curve, *Sen* sensitivity, *Spe* specificity, *PPV* positive predictive value, *NPV* negative predictive value, *Acc* accuracy

### Predictive nomogram for the probability of metastatic LNs in patients with PTC

**Fig. 4:**
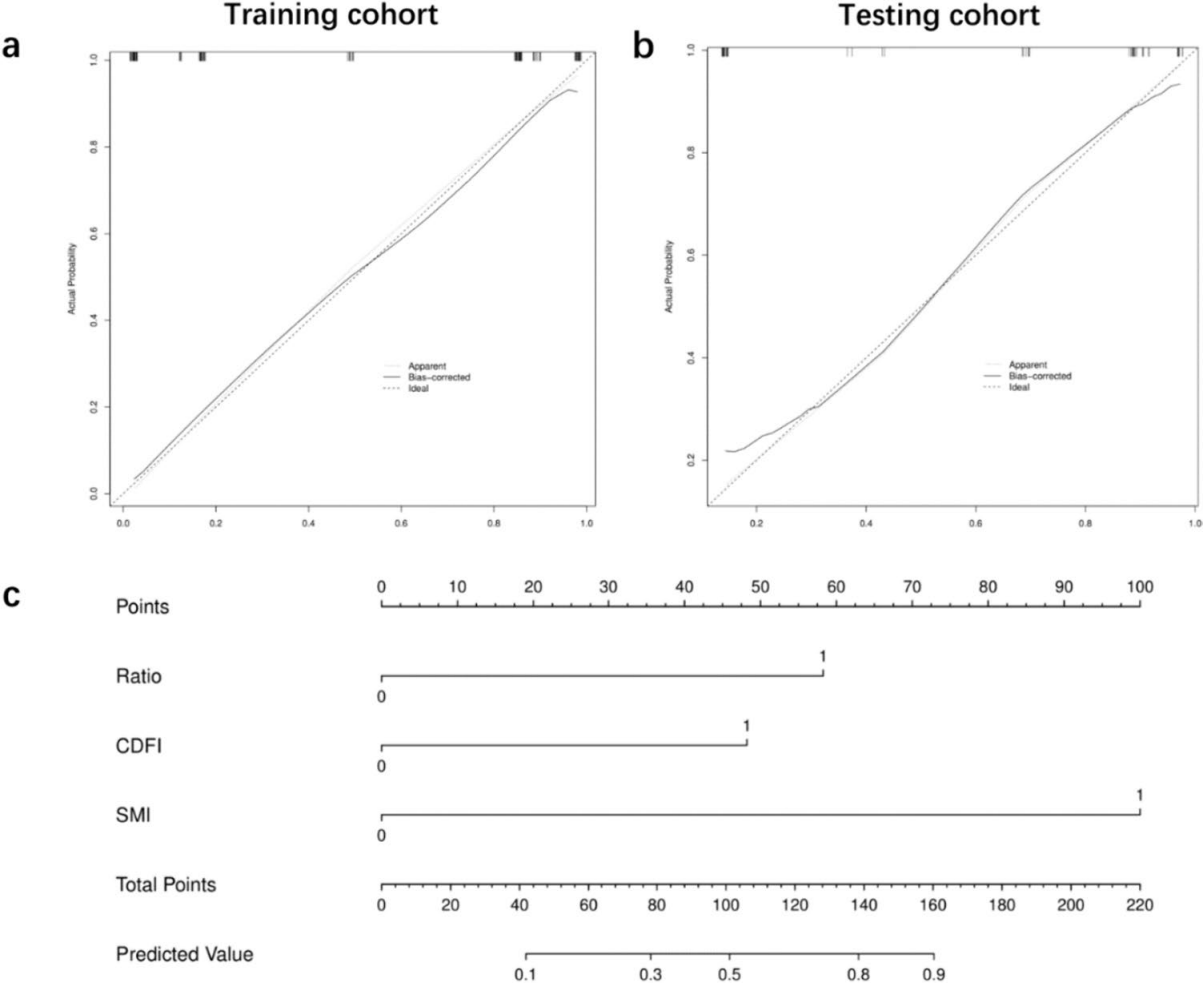
**a**, **b** Calibration curve for the nomogram in training and testing cohort; **c** nomogram for predicting the probability of metastatic LNs in patients with PTC. Ratio 0: L/S ≥ 2, 1: L/S < 2; CDFI 0: avascular or hilar type, 1: peripheral or mixed type; SMI 0: avascular or hilar type, 1: peripheral or mixed type

A nomogram was built based on multivariate regression analysis. Independent predictors for metastatic LNs, including L/S < 2, peripheral or mixed vascular type in CDFI, and peripheral or mixed vascular type in SMI, are shown in the nomogram (Fig. 4).

The calibration curves corresponded well with the prediction results and observations in the validation dataset (Fig. 4).

### Special vascular characteristics of SMI

**Fig. 5:**
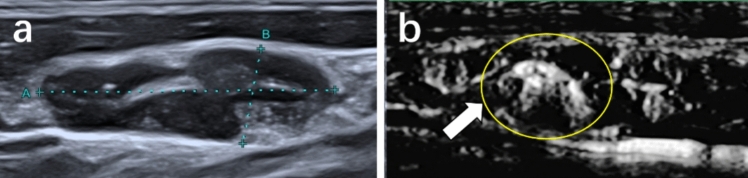
Metastatic lymph node from a 39-year-old man. **a** Conventional ultrasound. **b** Superb microvascular imaging (SMI), the lymph node exhibits focal abundant vessel distribution

**Fig. 6:**
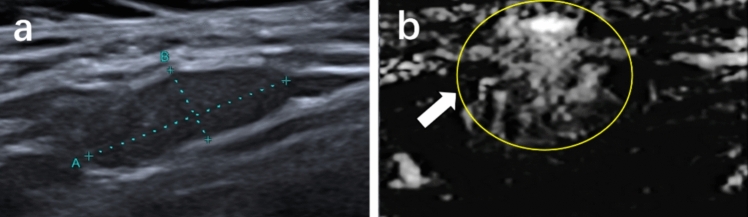
Metastatic lymph node from a 48-year-old woman. **a** Conventional ultrasound. **b** Superb microvascular imaging (SMI), the lymph node exhibits misty vessel distribution

**Fig. 7:**
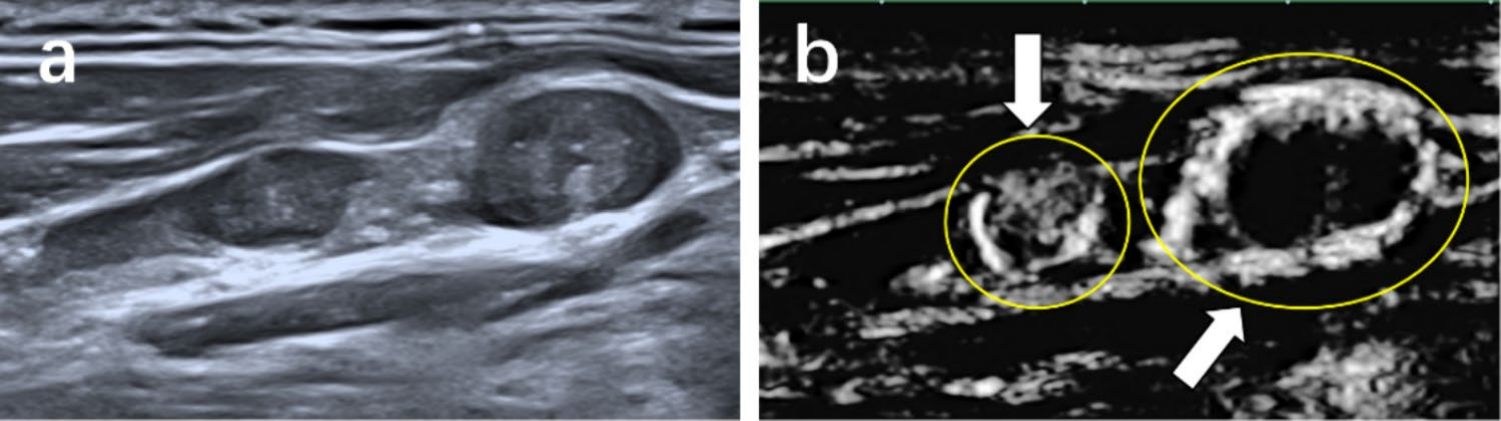
Metastatic lymph node from a 39-year-old man. **a** Conventional ultrasound. **b** Superb microvascular imaging (SMI), the lymph node exhibits ring-shaped vessel distribution

Based on our observations, focal abundant performance (Fig. 5), misty flow performance (Fig. 6), and ring-shaped flow (Fig. 7) of the vascular distribution emerged frequently in metastatic LNs on SMI. Significant differences were found between the two groups in the aforementioned vascular characteristic (*P* < 0.05), as shown in Table 4.

**Table 4:** Special vascular characteristic of SMI

Vascular pattern	Metastatic LN	Reactive LN	*P* value
Focal abundance or misty flow			0
Present	29 (28.2%)	3 (3.8%)	
Absent	74 (71.8%)	77 (96.2%)	
Ring-shaped flow			0
Present	20 (19.4%)	2 (2.5%)	
Absent	83 (80.6%)	78 (97.5%)	

*SMI* superb microvascular imaging, *LN* lymph node

## Discussion

In this study, we demonstrated that SMI can indicate characteristic blood flow distribution of metastatic LNs with PTC, improving the preoperative diagnostic accuracy of metastatic LNs. Compared with US alone, SMI combined with US improved accuracy, specificity, and PPV, with AUC of 0.829 and 0.926, respectively.

Vascularity was considered an important characteristic in metastatic cervical LNs with PTC, which was confirmed by pathological evidence in a recent study showing that microvessel density and microvessel area in the metastatic LN group were significantly higher than those in the non-metastatic LN group (Liu and Li 2022). Similarly, in this study, the SMI vascularity index between level 2–3 in metastatic LNs (56%) was significantly higher than that in reactive LNs (23%) (Table 1). Therefore, we suppose that compared to US, SMI can optimize the diagnosis of metastatic LNs.

Angiogenesis is essential for tumorigenesis, proliferation and invasion (Folkman et al. 1971). The particular vascular distribution in malignant LNs is related to neovascularization, desmoplastic reaction, and capsule vessel proliferation. Tumor cells enter the LNs through afferent lymphatic vessels, cell proliferation leads to cortex enlargement, and tumor cells invade the remaining normal tissue (Bonacchi et al. 2017). Hence, peripheral and mixed vascular distribution was present in most malignant LNs, which was consistent with the results of Sim et al. (Sim et al. 2019; Ryoo et al. 2016; Seongyong et al. 2020), and the hilar vascular pattern was the most common in normal or reactive LNs (Ahuja and Ying 2003). Similarly, in this study, most metastatic LNs exhibited a peripheral mixed vascular pattern (94%) and reactive LNs mainly exhibited avascular or hilar vascular pattern (79%). Notably, we found several special vascular characteristics of SMI emerging in partially metastatic LNs but rarely seen in reactive LNs, as mentioned above (see “Special vascular characteristics of SMI”). Consistently with this, our previous studies reported that metastatic LNs manifested heterogeneous enhancement and ring-enhancing margins more often than benign LNs on preoperative CEUS (Hong et al. 2017; Luo et al. 2022). We believe that this special vascular distribution is related to the pathological mechanism of tumor formation. The ring-shaped vascular distribution is likely due to metastatic cells entering LNs through afferent lymphatic vessels and spreading from the marginal sinuses (Ji et al. 2020; Fengkai et al. 2022). The focally abundant and misty flow performance are due to rapid cell proliferation and immature neovascularization (Fengkai et al. 2022). In addition, the indolent characteristic of PTC result in incomplete invasion of cervical LNs by tumor cell, which also contributes to focal abundance and misty flow performance.

With the emergence of SMI in 2014, based on the Canon medical system (Ohno et al. 2017), many studies have proven its ability to diagnose human diseases, such as tumors, inflammation and injury, owing to visualization of small vessels with slow flow in tissues (Ahuja and Ying 2003; Bae et al. 2020; Sato et al. 2021; De Backer et al. 2013). CEUS is a promising tool for providing capillary vessel perfusion information (Hong et al. 2017; Fengkai et al. 2022). A previous study proved that the diagnostic value of SMI was comparable with CEUS in thyroid nodules (Zhao et al. 2020).

Furthermore, a nomogram based on the training dataset was conducted to evaluate the predictive value of SMI and US for metastatic LNs with PTC. Parameters with statistical significance in the univariate analysis were selected for multivariate analysis. The results revealed that L/S < 2 and peripheral or mixed vascular type on CDFI and peripheral or mixed vascular type on SMI were independent predictors of metastatic LNs in patients with PTC. Peripheral or mixed vascular type on SMI had the highest predictive value with the highest odds ratio (OR 40.829), compared to L/S < 2 (OR 8.684) and mixed vascular type on CDFI (OR 5.982).

This study had several limitations. First, a potential selection bias is inevitable in this retrospective study. SMI was not a routine examination in the clinic practice, and partially reactive LNs which underwent SMI were considered suspicious on US. Second, the patients were enrolled at a single center; observer bias was present in this study, and a multicenter study with a larger number of patients is required. Third, the imaging findings of the vascular structures did not correlate with the true specimen pathology. Further research on pathological vessel distribution corresponding to SMI imaging is needed.

In conclusion, our findings demonstrate that SMI can significantly improve the diagnostic accuracy of metastatic cervical LNs from PTC. We also established and validated an accurate nomogram for predicting the possibility of LN metastasis in patients with PTC. Preoperative SMI has the significant potential to enhance the ability of surgeons to determine optimal clinical management.

### Supplementary Information

Below is the link to the electronic supplementary material.

Supplementary file1 (DOCX 193 KB)

## Data Availability

The data that support the results of this study are available from the corresponding author on reasonable request.
