# Supplementary material for: Use superb microvascular imaging to diagnose and predict metastatic cervical lymph nodes in patients with papillary thyroid carcinoma
Source: J Cancer Res Clin Oncol. 2024 May 21;150(5):268. doi: 10.1007/s00432-024-05770-x (PMC11108919; doi:10.1007/s00432-024-05770-x)
Supplement: Supplementary file 1 — Supplementary file1 (DOCX 193 KB) [file 432_2024_5770_MOESM1_ESM.docx]

**Supplementary materials**

**Table S1 Baseline clinical information of all cervical LNs in PTC**

| **Parameters** | **Training cohort(n=129)** | **Testing cohort(n=54)** | ***P* value** |
| --- | --- | --- | --- |
| Sex |  |  | 0.237 |
| Male | 39 (30) | 11 (20) |  |
| Female | 90 (70) | 43 (80) |  |
| Age | 38±12 | 37±12 | 0.685 |
| Location |  |  | 0.172 |
| Lateral | 95(74) | 41(76) |  |
| Central | 34(26) | 13(24) |  |
| Size(mm) | 11.86±4.74 | 14.07±5.65 | 0.044 |
| Ratio(L/S) |  |  | 0.213 |
| ≥2 | 79 (61) | 39 (72) |  |
| ＜2 | 50 (39) | 15 (28) |  |
| Shape |  |  | 0.559 |
| irregular | 77 (60) | 29 (54) |  |
| Oval | 52 (40) | 25 (46) |  |
| Border |  |  | 0.761 |
| Ill-defined | 55 (43) | 21 (39) |  |
| Well-defined | 74 (57) | 33 (61) |  |
| Hilum |  |  | 0.814 |
| Present | 104 (81) | 42 (78) |  |
| Absent | 25 (19) | 12 (22) |  |
| Echogenicity |  |  | 0.577 |
| Isoechoic or hyperechoic | 72 (56) | 27 (50) |  |
| Hypoechoic | 57 (44) | 27 (50) |  |
| Homogeneity |  |  | 0.959 |
| Homogeneous | 50 (39) | 20 (37) |  |
| Heterogeneous | 79 (61) | 34 (63) |  |
| Cystic necrosis |  |  | 0.672 |
| Present | 5(4) | 1(2) |  |
| Absent | 124(96) | 53(98) |  |
| Calcification |  |  | 0.186 |
| Absent or macrocalcification | 104 (81) | 38 (70) |  |
| Microcalcification | 25 (19) | 16 (30) |  |
| CDFI vascular pattern |  |  | 0.457 |
| Avascular or hilar type | 67 (52) | 32 (59) |  |
| Peripheral or mixed type | 62 (48) | 22 (41) |  |
| CDFI vascular index |  |  | 0.065 |
| 0 | 30 (23) | 14 (26) |  |
| 1 | 84 (65) | 32 (59) |  |
| 2 | 13 (10) | 3 (6) |  |
| 3 | 2 (2) | 5 (9) |  |
| SMI vascular pattern |  |  | 0.933 |
| Avascular or hilar type | 50 (39) | 22 (41) |  |
| Peripheral or mixed type | 79 (61) | 32 (59) |  |
| SMI vascular index |  |  | 0.950 |
| 0 | 5 (4) | 2 (4) |  |
| 1 | 71 (55) | 30 (56) |  |
| 2 | 35 (27) | 13 (24) |  |
| 3 | 18 (14) | 9 (16) |  |
| Class |  |  | 0.718 |
| Reactive LNs | 58 (45) | 22 (41) |  |
| Metastatic LNs | 71 (55) | 32 (59) |  |

Note: LN, lymph node; PTC, papillary thyroid carcinoma; CDFI, color doppler flow imaging; SMI, superb microvascular imaging

**Fig.S1**


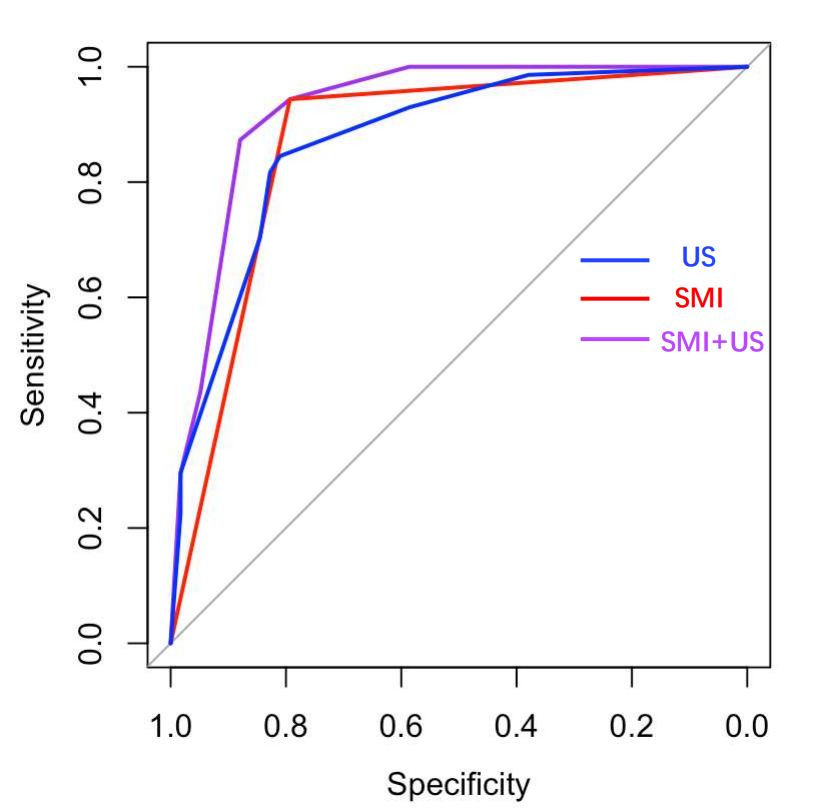


ROC curve analysis for predicting cervical lymph node metastasis in training cohort. AUC for SMI combined with US was the largest (AUC: 0.926), the sensitivity and specificity were 87.3% and 87.9% respectively; the sensitivity and specificity of SMI were observably higher than US (94.4%&87.3%; 79.3&69.3%, respectively). AUC: area under curve.
